# A Systematic Review and Bibliometric Analysis of Flame-Retardant Rigid Polyurethane Foam from 1963 to 2021

**DOI:** 10.3390/polym14153011

**Published:** 2022-07-25

**Authors:** Ying Pan, Chengliang Yin, Carlos Fernandez, Li Fu, Cheng-Te Lin

**Affiliations:** 1 College of Materials and Environmental Engineering, Hangzhou Dianzi University, Hangzhou 310018, China; panying@hdu.edu.cn; 2 National Engineering Laboratory for Medical Big Data Application Technology, Chinese PLA General Hospital, Beijing 100853, China; yinchengliang@301hospital.com; 3 Medical Big Data Research Center, Medical Innovation Research Division of PLA General Hospital, Beijing 100853, China; 4 School of Pharmacy and Life Sciences, Robert Gordon University, Aberdeen AB10 7GJ, UK; c.fernandez@rgu.ac.uk; 5 Key Laboratory of Marine Materials and Related Technologies, Zhejiang Key Laboratory of Marine Materials and Protective Technologies, Ningbo Institute of Materials Technology and Engineering (NIMTE), Chinese Academy of Sciences, Ningbo 315201, China; linzhengde@nimte.ac.cn

**Keywords:** rigid polyurethane, bibliometrics, flame retardant, expandable graphite, thermal degradation, composite

## Abstract

Flame-retardant science and technology are sciences developed to prevent the occurrence of fire, meet the needs of social safety production, and protect people’s lives and property. Rigid polyurethane (PU) is a polymer formed by the additional polymerization reaction of a molecule with two or more isocyanate functional groups with a polyol containing two or more reactive hydroxyl groups under a suitable catalyst and in an appropriate ratio. Rigid polyurethane foam (RPUF) is a foam-like material with a large contact area with oxygen when burning, resulting in rapid combustion. At the same time, RPUF produces a lot of toxic gases when burning and endangers human health. Improving the flame-retardant properties of RPUF is an important theme in flame-retardant science and technology. This review discusses the development of flame-retardant RPUF through the lens of bibliometrics. A total of 194 articles are analyzed, spanning from 1963 to 2021. We describe the development and focus of this theme at different stages. The various directions of this theme are discussed through keyword co-occurrence and clustering analysis. Finally, we provide reasonable perspectives about the future research direction of this theme based on the bibliometric results.

## 1. Introduction

With the rapid development of the social economy and urbanization, high-rise buildings have emerged in the city. At the same time, however, building energy consumption has become an important part of society’s energy consumption. Therefore, the development of energy-saving buildings has become the consensus of today’s society. Organic insulation materials are widely used worldwide due to their advantages, such as low density and excellent insulation properties [1,2]. However, most common organic exterior insulation materials available today have porous structures and huge specific surface areas that are very flammable [3,4,5]. Once ignited, the flame spreads rapidly, while many toxic gases are produced with significant fire hazards. Therefore, the research and development of fire-safe exterior wall insulation materials and systems to reduce building fire hazards have become important themes in today’s urban public safety and building development. Currently used organic exterior insulation materials are mainly rigid polyurethane foam (RPUF), expanded polystyrene (EPS), extruded polystyrene (XPS), and phenolic foam (PF) [6,7,8].

Polyurethane (PU) is a class of polymer compound containing carbamate (-NHCOO-)-repeating units in the main chain, generally prepared by polyorganic isocyanate and polyol reaction. This class of polymer was first synthesized by the German chemist Otto Bayer and his colleagues in 1936. After theoretical research, industrial processes, and application research, the PU industry has developed rapidly in the subsequent development. PU materials with different structures and properties can be prepared using raw materials with different types and numbers of functional groups [9,10]. Among them, RPUF is produced by the reaction of polyol and polyisocyanate, which generally accounts for more than 80% of the total mass of RPUF [11].

The excellent performance of RPUF cannot be achieved without additives. The additives are flame retardants, catalysts, surfactants, and foaming agents [12]. In the preparation of RPUF, the following chemical reactions exist simultaneously: (1) foaming reaction between isocyanate and water; (2) reaction between isocyanate and hydroxyl-containing compounds; (3) trimerization reaction between isocyanates; (4) chain expansion reaction of amine-based compounds; and (5) diuret reaction and ureidoformate reaction. In the presence of a catalyst, these reactions proceed simultaneously at a fast rate, in some cases, most of the reaction takes place with a few minutes, and, finally, a highly cross-linked RPUF is prepared.

RPUF has excellent thermal insulation properties with a closed-cell ratio of over 90% and low thermal conductivity of the air inside its bubble pores. Therefore, RPUF is a good adiabatic insulation material, even if the thickness is very thin [13,14,15]. RPUF has good mechanical strength and still has good dimensional stability at low temperatures. Specifically, when RPUF was kept at −20 °C for 24 h, the linearity of change was less than 1%. RPUF also has excellent aging resistance and long insulation life. Practical applications showed that RPUF can be used at −190–70 °C for up to 14 years. While the use of RPUF is growing rapidly, it also faces some pressing problems. The limiting oxygen index (LOI) of RPUF without flame-retardant treatment is only 18%. It is very easy to ignite and burns quickly, and, in the process of combustion, it releases hydrogen cyanide, carbon monoxide, and other toxic gases, accompanied by smoke [16]. With the gradual expansion of RPUF applications, potential fire hazards during use must also be considered. Therefore, it is of great practical importance to give RPUF flame-retardant properties and low toxicity by treating it with a flame-retardant treatment [17,18].

Several scientists provided excellent summaries of RPUF [19,20,21,22]. For example, a review of the state-of-the-art design of RPUF was conducted by Zhu et al. [21]. They summarized the reactive-, additive-, and coating-type flame retardants for RPUF. In addition, they compared the performance of different RPUFs. The use of bibliometrics allows for a kind of statistically based analysis of the evolution of a topic. This review summarizes the development of flame-retardant RPUF. This review attempts to summarize and address the following questions using bibliometrics:

(1) Which countries/companies have made important contributions to the development of flame-retardant RPUF? What led them to devote themselves to this theme?
(2) Is there an extensive international exchange of cooperation on the theme of flame-retardant RPUF?
(3) What types of flame retardant can improve the flame retardancy of RPUF? What is their flame-retardant mechanism?
(4) Have the rapid developments in materials science in recent years had an impact on the research on this theme?
(5) Are the flame-retardant properties that RPUF exhibits in the laboratory applicable when used as a building insulation material?

## 2. Methods

**Figure 1:**
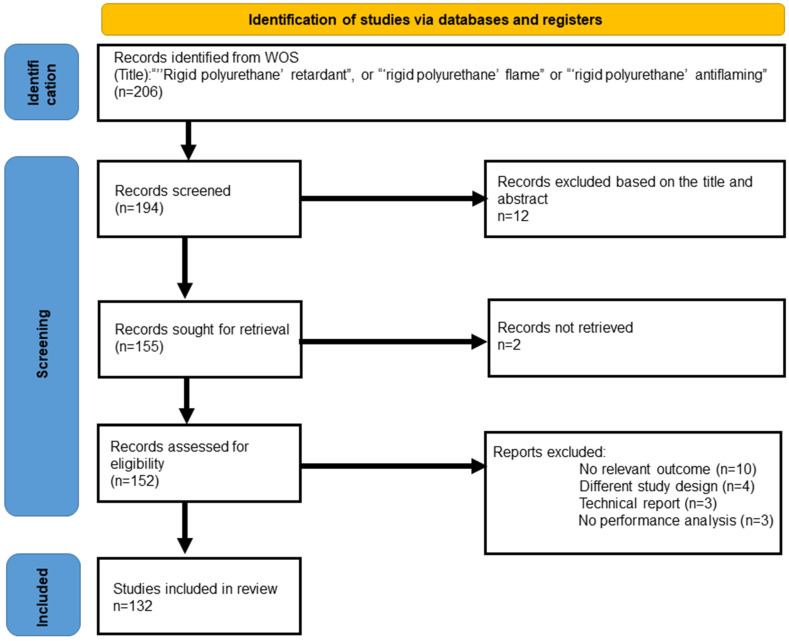
The preferred reporting items for systematic reviews and meta-analyses (PRISMA) for this study.

Two pieces of bibliometrics software were used in this systematic literature review. The first was CiteSpace, developed by Dr. Chaomei Chen, a professor at the Drexel University School of Information Science and Technology [23,24,25,26]. It has become one of the commonly used pieces of software in bibliometrics analysis. CiteSpace 5.8R3 was used to calculate and analyze all documents. COOC is another emerging piece of bibliometrics software [27]. COOC12.6 was used to calculate and analyze all documents. We used the core collection on Web of Science (SCI-Expanded) as a database to assure the integrity and academic quality of the studied material. “‘Rigid polyurethane’ retardant”, “‘rigid polyurethane’ flame”, and “‘rigid polyurethane’ antiflaming” were used as a “title”. The retrieval period was indefinite, and the date of retrieval was 30 December 2021. A total of 194 articles were retrieved, spanning the years from 1963 to 2021. The detailed systematic literature search (preferred reporting items for systematic reviews and meta-analyses, PRISMA) from the Scopus database is shown in Figure 1.

## 3. Results and Discussion

### 3.1. Developments in the Research Field

#### 3.1.1. Literature Development Trends

**Figure 2:**
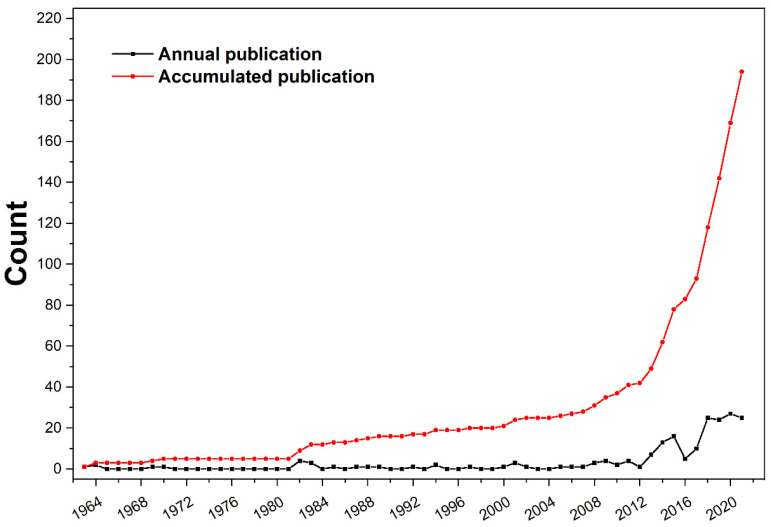
Annual and accumulated publications from 1963 to 2021 searched in the Web of Science about flame-retardant RPUF.

The number of papers published is an important indicator used to measure whether a theme is widely attracting the attention of scholars. Especially for a theme with a long history, analyzing the number of papers published can give an idea of whether the field has seen breakthroughs in its development. Figure 2 shows the annual and cumulative publications on flame-retardant RPUF from 1963 to 2021. As can be seen from the figure, the earliest published record on this theme dates back to 1963. James J. Anderson from the Virgina-Carolina Chemical Company investigated the physical and chemical factors that affect the flame retardancy of RPUF [28]. His investigation found that the key to improving the flame resistance of RPUF was to reduce the amount of phosphorus in the additive. The second-ranked factor was the internal structure of the RPUF, although this was directly related to the type of polyol used. The following year, two more papers on RPUF were published, proposing the preparation of RPUF using chlorinated xylene derivatives [29] and monobrominated toluene diisocyanate [30], respectively. For a long time afterward, sporadic publications were devoted to synthesizing novel RPUF and the corresponding property determination. It is worth noting that the publication of papers in this very early stage was led by chemical companies rather than based in universities or national academic institutions. On the other hand, the core collection on Web of Science started to include papers in 1990. Although it retroactively included data from before 1990, not all papers on RPUF are included in the database due to the rules of searching journals. However, according to the trend shown in Figure 1, there was no continuous year-by-year publication on this theme until the beginning of 2005. Therefore, data prior to 1990 do not affect the main conclusions drawn from the bibliometric analysis of this theme. In the course of history, the annual number of academic papers presented a rising trend. In addition to the increase in the total number of academic papers published, the number of researchers and academic topics also showed a rising trend. However, there is no effective model that can be used as a background line for contrasting the difference between a topic’s growth and its historical background. Therefore, when we use bibliometrics to discuss a theme, we still analyze whether a theme has received attention from the academic community by the annual publication number.

Starting from 2005, this theme started to be featured in publications every year, but the number of annual publications was below five until 2013. Starting in 2013, the number of papers on flame-retardant RPUF began a marked upswing and briefly reached a peak (16 papers) in 2015. Within this period, intumescent, flame-retardant RPUF received special attention. In particular, in 2013, more than half of the papers focused on the investigation of intumescent, flame-retardant RPUF [31,32,33,34]. This trend was not consistently maintained, with the annual number of publications showing a significant decrease in 2016 and 2017, with 5 and 10 publications, respectively. However, this theme started to attract much attention once again in 2018. So far, the annual publication rate has remained at more than 24 papers. From these historical data, it can be seen that flame-retardant RPUF had a brief research boom between 2013 and 2015. After two years of silence, the theme has returned to the forefront and has remained hot.

#### 3.1.2. Journals, Cited Journals, and Research Subjects

**Figure 3:**
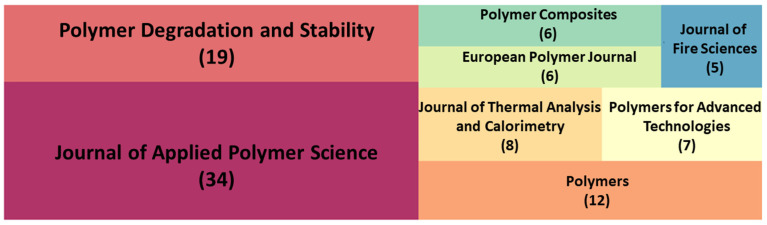
Top 8 journals that published articles on flame-retardant RPUF.

Figure 3 shows a tree diagram of the top eight journals that have published a number of flame-retardant-RPUF-related papers. The Journal of Applied Polymer Science has published an unexpectedly large number of papers on this theme. Specifically, more than a quarter of the papers on this theme were published by this journal. This phenomenon is also relatively rare in bibliometric surveys. This may be because flame-retardant RPUF is a material directly related to industrial applications. Therefore, the relevant papers were focused on measuring its functional properties rather than limited to theoretical base studies. The Journal of Applied Polymer Science is a journal that publishes the properties of polymeric materials for different applications, making it the most important journal relating to this theme. In addition, the main content on this theme investigates the flame-retardant properties of RPUF, so the thermal stability of RPUF and the processes and products of decomposition were also important targets for investigation, which can explain the second-ranked journal being Polymer Degradation and Stability. In addition, a significant share belonging to polymer-related journals can be seen in Figure 3. The remaining journals also include a journal related to thermodynamics (Journal of Thermal Analysis and Calorimetry) and a flame-science-related journal (Journal of Fire Sciences). The scopes of these two journals are also directly related to this theme.

**Figure 4:**
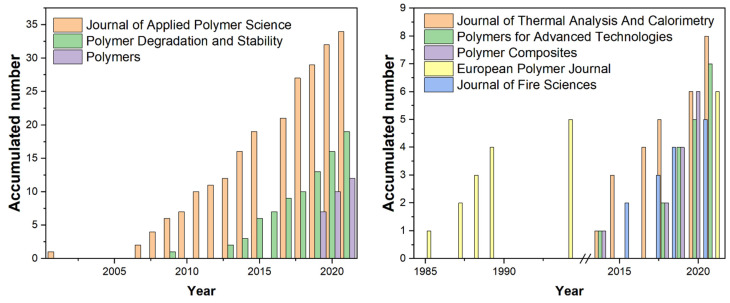
Time-dependent, cumulative number of publications of journals in Figure 3 related to flame-retardant RPUF.

Figure 4 shows the cumulative number of publications on this topic in the journals in Figure 3 at different times. Among them, the European Polymer Journal published a series of papers on this topic before 1994, which was the earliest among all the journals shown in Figure 3. In addition, the Journal of Applied Polymer Science published a series of papers on this topic before 2010. Most of the other journals in Figure 3 published papers on the theme after 2010. However, the analysis shown in Figure 4 had some limitations. This is because journals are launched in a sequential order. Even classic journals change their names for a number of reasons. Other journals cease, making it impossible to measure their contribution to a theme statistically. However, journal analysis can give some hints on a theme’s publishing strategy. Using flame-retardant RPUF as an example, it can be seen that polymer-related journals were preferred for this theme. Journals focusing on thermodynamics and fire science were also worth considering.

In addition to the number of papers on the theme published by the journal, the frequency with which the journal is cited by papers related to the theme is also an important indicator. Table 1 shows the top 15 cited journals on flame-retardant RPUF. The top three journals and the order are consistent with the journals in Figure 3, representing that these journals published a large number of papers in this theme and were also the most cited journals in this theme. All eight journals in Figure 3 are included in Table 1, representing the significant role these journals played in advancing this theme. In addition, not surprisingly, some comprehensive materials science journals were often cited, such as Fire and Materials and Composites Science and Technology. It is worth noting that the appearance of the two journals did not follow the pattern derived in Figure 3. The first journal is the fifth-ranked Industrial & Engineering Chemistry Research. This is a journal that is very well known in chemical engineering. RPUF’s obvious value can explain its presence and high ranking for industrial applications. It can even be said that the development of research on this theme was led by the need for industrial development (based on the conclusions drawn from the publication of papers on this theme at an early stage in Figure 2). The second journal is Industrial Crops and Products, ranked 12th, and it is not easy to explain the presence of agriculture-related journals with a high ranking in this theme. We found two highly cited papers related to RPUF in this journal [35,36]. They both used castor oil as a polyol for the synthesis of RPUF. Because of the relationship between castor oil and crops, the journal published both papers and received considerable attention on this theme.

**Figure 5:**
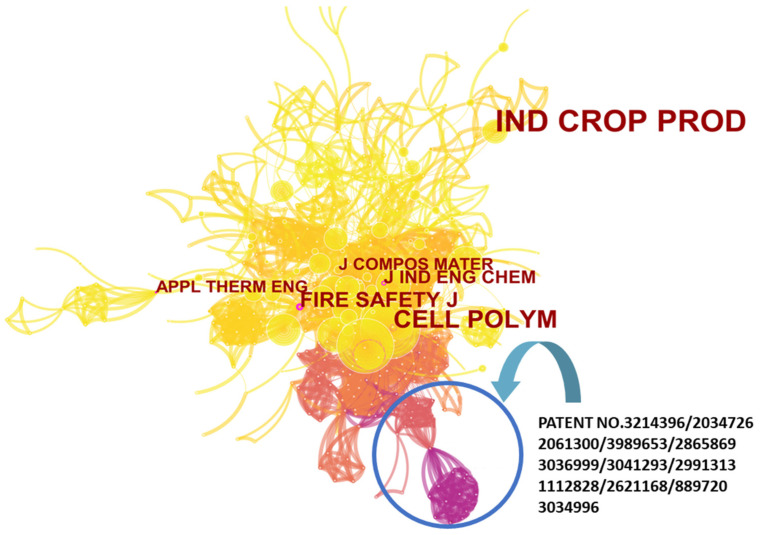
Co-occurrence network of cited journals for flame-retardant RPUF.

To further explore the information that journals can provide, we constructed a co-occurrence network of cited journals related to flame-retardant RPUF (Figure 5). In this figure, we have not labeled most of the journals that were discussed in Figure 3 and Table 1, except for Industrial Crops and Products. The figure shows that Industrial Crops and Products, although it had many citations (large radius of the node), is not in the center of the co-occurrence network, which means that the paper on flame-retardant RPUF published in Industrial Crops and Products was not cited by the most published series of journals on this theme. This often occurs when work on one theme is cross-researched with other areas. A very similar situation in Figure 5 is also seen for Applied Thermal Engineering. At the center of the co-occurrence network are some other journals that significantly impacted this theme but are not listed in Figure 3 and Table 2, such as Cellular Polymers, the Journal of Composite Materials, the Journal of Industrial Engineering Chemistry, and the Fire Safety Journal. Although these journals have not published many papers on this theme or are highly cited, they link to a very large number of nodes that represent significant contributions to the development of this theme. In addition, the co-occurrence network of cited journals of this theme has a distinct feature in that it contains a large number of patents (the lower part of the network presents dark nodes and lines). The sub-network formed by these patents further represents the solid industrial background of the theme.

Table 2 shows which cited journals this theme was extended to for the first time in 2020 and 2021. Based on the data in Figure 2, it is known that this theme was at the peak of publication in the last four years, and, therefore, it extended to many journals. Most of the journals in Table 2 are material-science- and chemistry-related journals. These journals have a direct and more intimate connection to the theme. In addition, this theme is beginning to receive more attention in environmental journals, such as Environmental Science & Technology Letters, Reviews on Environmental Health, Austral Ecology, etc. This may be because RPUF’s environmental impact is starting to be taken seriously. In 2021, cited journals on this theme also included journals on biology and food science, further representing that the toxicological properties of RPUF may also affect food safety and biosafety. It is worth noting that the co-occurrence network of cited journals on this theme was also extensively involved in international standards and reports in recent years. We have not encountered this phenomenon when analyzing other themes using bibliometrics [37,38,39,40,41], further illustrating that this theme, although in a research boom at this stage, already has an extensive range of applications in the industry.

**Figure 6:**
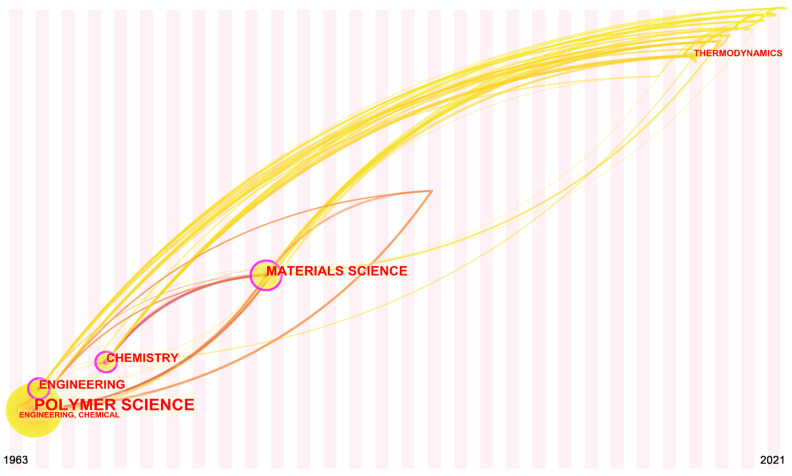
Time-zone view of research categories for flame-retardant RPUF.

The category of the published paper can reflect the evolution of the theme. Figure 6 shows the evolution of the category of the flame-retardant RPUF over time. As shown in the figure, there is an obvious path developing in the category for this theme. In the early stages of the theme, engineering, chemical engineering, chemistry, and polymer science led the way. This is because RPUF synthesis is directly related to these fields. At present, the main methods for preparing RPUF in industrial production are the prepolymer method, semi-prepolymer method, and one-step method [42]. The prepolymer method reacts polyester or polyether with polyisocyanate to form prepolymer and then mixes the formed prepolymer with a catalyst, foaming agent, and other preparation materials to form RPUF. The semi-prepolymer method is the reaction of polyester polyols or polyether polyols with excess polyisocyanate to form prepolymer intermediates. The end of the prepolymer intermediate contains isocyanate group and then the formed prepolymer intermediate, polyester polyol, or polyether polyol is mixed with the preparation materials such as polyisocyanate, catalyst, and foaming agent to form RPUF [43]. The one-step method mixes polyester polyol or polyether polyol, polyisocyanate, a catalyst, a foaming agent, and other preparation materials to directly foam and generate RPUF in one step. Starting in 1983, this theme began to enter materials science. This is because inorganic fillers are beginning to be widely used to improve the flame retardancy of RPUF. Inorganic fillers generally have large specific heat capacity; heat conduction can also be heat storage. Therefore, they can control the temperature of the polymer matrix, and it is not easy for them to reach the thermal decomposition limit [44]. In 2014, this theme began to rapidly expand into other categories, including agriculture, agronomy, and thermodynamics. The papers on agriculture and agronomy were on the previously mentioned use of castor oil as a polyol to synthesize flame-retardant RPUF and test its performance [35,36]. On the other hand, the discovery of the thermodynamics field played a very important role in its subsequent development. Gao et al. [45] investigated the thermal degradability and flame retardancy of RPUF containing intumescent flame retardants. Papers on this theme were gradually published in thermodynamics-related journals from this work. Material flame-retardant performance test methods can generally be divided into six categories: (1) ignition and flammability (such as ignition temperature and limiting oxygen index); (2) flame propagation (such as tunnel test and radiation plate test); (3) heat release (such as cone calorimeter and calorimeter test); (4) smoke generation (such as smoke box test and soot quality test); (5) combustion product toxicity (such as biological tests and chemical analysis method); and (6) flame resistance (such as building components fire resistance test).

In 2018, this theme entered the category of forestry for the first time. Lu et al. [46] investigated the flame-retardant properties of lignosulfonate-based RPUF. Lignin is considered an excellent carbonizing agent because of its high aromatic ring content and high carbon content. Starting in 2019, environmental science and ecology started to focus on flame-retardant RPUF. For example, Xu et al. [47] tried to add diatomite with melamine-coated zeolitic imidazolate framework-8 to RPUF to improve its flame-retardant and smoke-suppressing properties. Overall, flame-retardant RPUF is a theme with a long history, but it does not intersect with too many fields in academic publications. Chemistry, engineering, polymers, and materials science are the most basic categories related to this theme. Some other categories involved recently are mainly the subcategories under these fields. Although some of these papers were published in journals related to other categories, their content did not present a solid interdisciplinary character.

#### 3.1.3. Geographic Distribution

**Figure 7:**
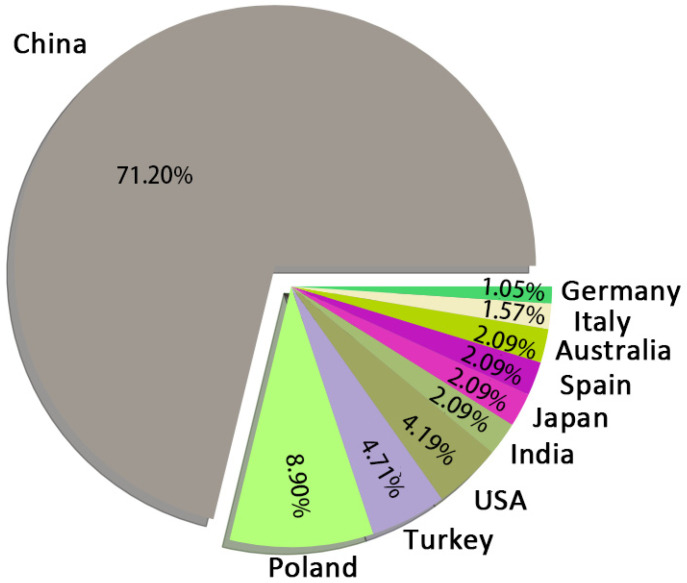
Pie chart of papers related to flame-retardant RPUF contributed by different countries.

Figure 7 shows the top 10 countries with the most publications on flame-retardant RPUF. China has contributed more papers on this theme than all other countries combined. This phenomenon may be linked to the massive demand for building insulation materials in China’s rapid urbanization process. Building insulation material is mainly used to keep the indoor temperature of buildings constant by reducing indoor heat emission and preventing outdoor cold penetration, which is generally achieved by applying insulation materials to the building envelope. Therefore, in areas with freezing weather, good building insulation material can effectively keep the temperature in the building fluctuating within a specific range, which can reduce the heating and energy consumption. Developing building insulation materials with good insulation performance, low cost, and efficient stability maintenance performance has become a hot spot in Chinese scientific research. RPUF is widely used in building insulation projects because of its low density, light weight, low thermal conductivity, and high thermal storage coefficient. However, RPUF is a flammable material with poor flame-retardant properties, limiting its application as a construction material. Building fire incidents caused by RPUF occurred from time to time from 2008 to 2012. Therefore, scientists in China continued to modify RPUF for flame retardancy after 2013. They improved the flame-retardant properties of RPUF by adding various functional flame retardants and also published a large number of papers. In addition to China, Poland, Turkey, and the USA also made significant contributions to this theme, publishing 8.90%, 4.71%, and 4.19% of the papers.

**Figure 8:**
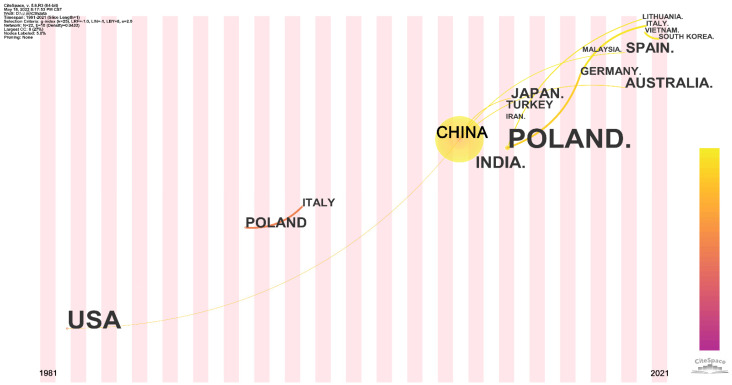
Time-zone view of geographic distribution for flame-retardant RPUF.

Figure 8 shows the time-zone view of the geographic distribution for flame-retardant RPUF papers. The earlier literature in the core collection on Web of Science did not include the published countries, so the time-zone view only contains the relationship between countries from 1982 to 2021. Links between different countries were established based on papers published in those countries being directly cited. Although China contributed the vast majority of papers on this theme, it did not become involved until 2008 and was influenced by a paper published by the USA in 1982. The paper published by China further prompted several other countries to engage in the theme, including Japan, Turkey, Spain, and Australia. Poland was involved in this theme in 1994 and triggered the Italian paper 4 years later. Then, it was not until 2011 that Poland published again on this theme and triggered the entry of Germany and Lithuania into the theme. In addition, a paper published by Vietnamese scientists in 2020 caused South Korea to engage with this theme in 2021.

**Figure 9:**
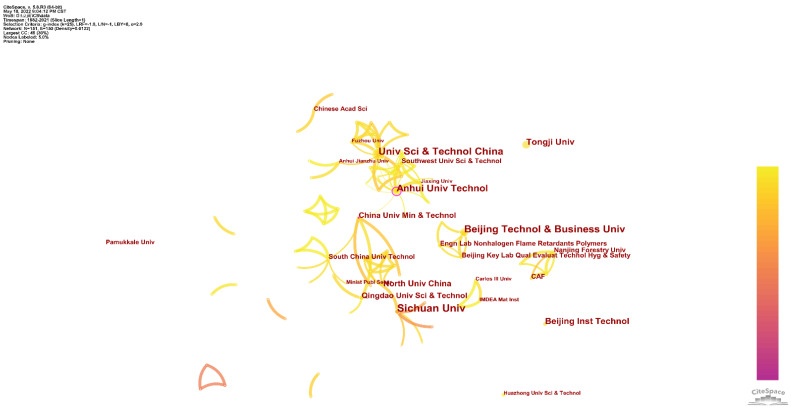
Institution cooperation network for flame-retardant RPUF.

Although not many countries are involved in this theme, there is more frequent cooperation between different institutions. Figure 9 shows the institutional cooperation network for this theme. There is a significant cooperative network dominating the scientific research on this theme. This large collaborative network can be further subdivided into two smaller sub-networks connected by the Anhui University of Technology and the China University of Mining & Technology. The University of Science and Technology of China leads the first sub-network. Important institutions include Fuzhou University, Anhui Jianzhu University, Southern University of Science and Technology, and Jiaxing University. The second sub-network is led by Sichuan University and the North University of China. Important institutions include the South China University of Technology, Tianjin Fire Research Institute of the Ministry of Public Security, and Qingdao University of Science and Technology. The theme also includes small cooperative networks, such as Beijing Technology and Business University, which has published many papers and leads a small cooperative network. In addition, some independent institutions have also made significant contributions to this theme. For example, Tongji University has eight published records.

### 3.2. Keyword Analysis and Evolution of the Field

The most effective way to understand the direction of investigation of concerns in a theme is the analysis of keywords. Table 3 lists the top 15 keywords in this theme. Since this theme is about the flame-retardant properties of RPUF, the most frequent keyword should be PU or flame retardant. However, it is interesting to note that the most frequent keyword was expandable graphite. Expanded graphite has a flaky graphite structure and is an intumescent, halogen-free flame retardant that excels in improving the flame-retardant properties of polymers [48,49,50,51,52]. The hexagonal stacked lamellar structure of the sp^2^ hybridization of expanded graphite is treated with acetic acid, sulfuric acid, or nitric acid, which is inserted into graphite crystals [53]. The flame retardancy of expanded graphite is mainly in the condensed phase, and it can also significantly reduce the smoke density during the combustion of polymers [54,55]. When the expanded graphite is heated, the inserted compounds, such as sulfuric acid, decompose which produces sulfur dioxide and water. On the other hand, the expanded graphite undergoes an oxidation reaction in the presence of an acid, such as the reaction in the presence of sulfuric acid to produce sulfur dioxide and carbon dioxide [56]. The released gas creates a large pressure between the lamellae, causing the spacing of the expanded graphite lamellae to increase sharply. This forms a worm-like, low-density insulation layer on the surface of the polymer matrix which can effectively block the transmission of heat and oxygen, thus, providing a good flame-retardant effect [57]. However, the addition of large amounts of expanded graphite to RPUF can lead to the deterioration of its mechanical properties. Synergistic flame-retardant or surface-modification methods can reduce the damage to mechanical properties of RPUF by expanded graphite [58]. This situation is also prevalent for other mechanically added flame retardants. This explains why mechanical property was the second most frequently occurring keyword. The keywords related to specific material properties in Table 3 include density and stability. On the other hand, thermodynamics-related terms and keywords for changes in material form also appear in Table 3, including fire behavior, thermal degradation, degradation, and combustion.

Phosphorus and ammonium polyphosphate were two high-frequency keywords (ranked fifth and sixth) because phosphorus-containing flame retardants are widely used in polymeric materials as a class of halogen-free flame retardants [59,60]. The retention of phosphorus in the condensed phase reduces the fire hazard, especially when it promotes the charring of polymers or the formation of inorganic glass-like layers. The presence of phosphorus accelerates the dehydration of the polymer, which leads to cyclization, cross-linking, aromatization, and graphitization. At the same time, the decomposition products of the phosphorus-containing compounds act as cross-linking agents [61,62]. Then, a charred layer (possibly an expanded char layer) is formed on the polymer surface. The presence of polyphosphates can also lead to a glassy inorganic layer [63]. This char or glassy layer can act as a physical barrier between the gas and condensed phases. Such a protective layer limits the transfer of combustible volatile gases and oxygen so that the gas yield from decomposition is significantly reduced. Moreover, the fuel gas is physically isolated from oxygen, preventing the continuation of the combustion process. In addition to the condensed phase mechanism, some phosphorus-containing compounds can also function in the gas phase with a mechanism similar to halogen-containing flame retardants [64,65,66]. It is believed that phosphorus–oxygen radicals play a significant role. In this case, hydrogen radicals and hydroxide radicals in the gas phase are replaced by less reactive radicals or become non-radicals through radical recombination. The disproportionation reactions and chain reactions of hydrocarbon oxidation in the gas phase are slowed down or interrupted. This process is called flame suppression and reduces heat production.

We conducted the burst detection of keywords, but only three valid keywords (density, polymer, and hydroxide) were obtained. The first two of these burst keywords appeared in 2008 and 2010, respectively, and both lasted for 7 years. This means that the research on flame-retardant RPUF was mainly focused on the polymer and its basic properties at that stage. The burst keyword hydroxide appeared in 2019 and continues to appear to this day. This can be explained by the fact that layered hydroxides have been widely used in recent years to improve materials’ flame-retardant and smoke-suppression properties. For example, Wang et al. [67] used expandable graphite wrapped in magnesium hydroxide nanosheets as a flame retardant for RPUF. The results showed that the residual carbon edge of expanded graphite could be sealed after combustion after magnesium hydroxide nanoflakes were encapsulated, which significantly enhanced the expansion performance. The reaction of magnesium hydroxide nanosheets and isocyanate functional groups improved the interfacial adhesion of expanded graphite to the RPUF matrix. Thus, the cell structure and storage modulus of the synthesized RPUF were significantly improved. Peng et al. [68] also investigated the effect of aluminum hydroxide/magnesium hydroxide on the pore structure, compressive stress, combustion properties, and thermal stability of RPUF. They found that the addition of aluminum hydroxide improved the performance of RPUF to be superior to that of the RPUF modified by magnesium hydroxide.

**Figure 10:**
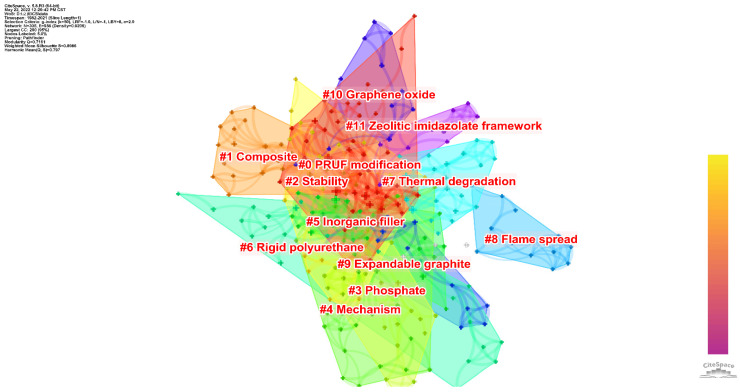
Grouping of keywords for flame-retardant RPUF.

Cluster analysis can further understand the different directions of investigation in this theme. Figure 10 shows that 12 clusters are formed after clustering the keywords. On the whole, many clusters have overlapping areas between them, indicating that their contents had more similarities with each other. Only one cluster does not appear to overlap with the other clusters. This also reflects that the theme of flame-retardant RPUF did not form a differentiation in different directions. Table 4 shows a detailed description of the clusters and their ID, size (number of papers), silhouette, and respective keywords. The following is a short explanation of each cluster.

# 0 RPUF modification: This cluster contains many works and has a high level of overlap with the regions of a series of other clusters. The main content of this cluster included the influence of different additives on the flame retardancy of RPUF, for example, fly ash [69], char [70], alumina trihydrate, triphenylphosphate [80], expanded vermiculite/melamine phenylphosphate composite [73], exfoliated clay [75], phosphoramide/expandable graphite [90], etc. Additional works contained other elemental additions of polyols for RPUF preparation and their effects, such as phosphorus and nitrogen-containing polyols [76,84]. Most of the work in this cluster is further classified in other clusters below;

# 1 Composite: The content of this cluster focused on improving RPUF flame-retardant properties by adding composite materials. For example, Xu et al. [47] synthesized diatomite/melamine-coated zeolitic imidazolate framework-8 composite material as an additive to improve RPUF. Ye et al. [102] synthesized an expandable graphite–poly(methyl methacrylate) composite to improve the flame-retardant performance of RPUF. Zhang et al. [103] chose to synthesize an expandable graphite–methyl methacrylate–acrylic acid composite to improve the flame-retardant performance of RPUF;

# 2 Stability: The region of this cluster is entirely covered by the regions of several other surrounding clusters, while its silhouette value is only 0.854, representing that the clustering homogeneity of the keywords is not particularly outstanding. This cluster contains 10 papers. These papers do not have a particularly uniform orientation in terms of subject matter. Some of the works focused on modifying phosphorus-containing materials [76,117] and others on the modification of ammonia-containing materials [112,113]. In addition to this, there were also some works on the detailed performance investigation of traditional RPUF [108,111]. The most frequently occurring keyword in this cluster was stability. All these works investigated the stability of the final, synthesized RPUF;

# 3 Phosphate: The content of this cluster was mainly concerned with the effects of using different phosphates on the properties of RPUF. For example, Hu et al. [117] used aluminum diethylphosphinate; Zhang et al. [118] chose castor oil phosphate. Generally speaking, there are two types of phosphorus-containing flame retardant: additive type and reactive type. The additive type is mainly used to disperse the flame retardant into the polymer matrix by physical mixing during the processing of the polymer material. This approach is more convenient and effective and is widely used in the industry. However, this approach has many disadvantages, such as easy migration and precipitation, poor compatibility with the polymer matrix, etc. The reactive approach mainly uses some reactive flame retardant to connect with the polymer matrix through a chemical reaction. Due to the chemical bond between the flame retardant and the polymer matrix, the material can maintain its flame-retardant properties for a long time. On the other hand, some works used bio-based materials; as well as castor oil phosphate, Ranaweera et al. [114] chose bio-based polyols;

# 4 Mechanism: The content of this cluster focused on the investigation of the fire behavior and flame-retardant mechanism of different RPUFs. Guenther et al. [121] investigated the morphological changes of RPUFs with different levels of combustion. They found that, as the foam density increases, the burning behavior of RPUF shifts towards a non-cellular material. Some other works focused on understanding the role of different additives when they are added to participate in flame-retardant behavior;

# 5 Inorganic filler: The addition of inorganic filler to improve the flame retardancy of RPUF was the focus of this cluster. A series of work focused on adding expanded graphite to improve the flame retardancy of RPUF [79,125,127,132,138]. Authors of other works chose alternative carbon materials. For example, Acuña et al. [128] incorporated both expanded graphite and graphene oxide. In addition to carbon materials, glass is also a very effective inorganic filler. Bian et al. [126] tried to improve the flame-retardant properties of RPUF with a hollow glass microsphere. Cheng et al. [131] investigated the difference between a hollow glass microsphere and glass fiber in improving the flame-retardant properties of RPUF;

# 6 Rigid polyurethane: This cluster is covered by the range of other clusters around it. There are 11 papers in this cluster. They all include the phrase “rigid polyurethane”, which is included in the title. However, they differ in their chosen methods and strategies to improve RPUF;

# 7 Thermal degradation: This cluster has the lowest silhouette value of all clusters (0.852), representing the low similarity of the papers in this cluster. A series of papers in this cluster focused on improving the flame-retardant properties of RPUF by expanded graphite [132,145,158,159,160,161]. Additionally, a series of works focused on improving modified phosphates for RPUF flame retardancy [75,81,132,134,145,161,162];

# 8 Flame spread: This cluster contains only two papers. The first paper investigated flame propagation behavior downward during RPUF combustion [150]. The authors provided a detailed qualitative analysis of the mechanism of the orientation effect during flame propagation based on experimental data. The other work investigated the upward flame propagation behavior during RPUF combustion [151]. These two works provide a very interesting discussion of the spatial propagation behavior of RPUF during combustion;

# 9 Expandable graphite: This cluster contains only five papers, and, also, its area overlaps with other surrounding clusters. All the papers in this cluster focused on the effect of expandable graphite on the performance of RPUF;

# 10 Graphene oxide: The additives chosen for the papers in this cluster were different from the other papers and included graphene oxide [163], steel slag powder [83], and N,N′-diethanolaminomethylphosphate [156];

# 11 Zeolitic imidazolate framework: Both works included in this cluster chose to use the zeolitic imidazolate framework for flame-retardant and smoke-suppression performance enhancement of RPUF.

Based on the clues given by the above cluster analysis, the research direction of flame-retardant RPUF can be briefly summarized as follows:

(1) Additives play a significant role in improving the flame retardancy of RPUF. Most of the works focused on enhancing the flame-retardant properties of RPUF using expanded graphite, phosphate, hydroxide, and ammonia-containing additives;
(2) Some new materials are also being tried to increase the flame retardancy of RPUF, such as graphene oxide and a zeolitic imidazolate framework. Additionally, preparing composites before adding them to RPUF is an option;
(3) This theme is mainly performance-oriented, and LOI, cone calorimetry, and thermogravimetric analysis are the most commonly used evaluation techniques;
(4) The flame-retardant properties of RPUF are related to the microstructure of RPUF and the nature of the additives;
(5) RPUFs containing different additives have different flame-retardant mechanisms;
(6) The burning behavior of RPUF toward different spatial regions during combustion is also a direction of investigation.

## 4. Conclusions

RPUF is an organic foam material that has attracted significant attention for its excellent thermal insulation properties, adhesion properties, specific strength, and durability. Currently, RPUF is widely used as a building exterior insulation material. However, it is highly flammable, which increases the risk of fire in construction materials, especially when used in high-rise buildings. Therefore, the research on the flame retardancy of RPUF is an important and urgent task. In this review, we analyzed 194 papers on the flame retardancy of RPUF between 1963 and 2021 using bibliometrics. The following conclusions are summarized from the analysis:

(1) The flame-retardant RPUF is a theme with a long history. Chemical companies published the earliest series of papers on this theme rather than universities and government-funded research institutions. As a result, the subject has a solid industrial application background. This feature has also made the theme largely relevant to the improvement of flame-retardant performance without the widespread impact of new material discoveries;

(2) Chinese scientists contributed many papers on this theme, probably because of the massive demand for building insulation materials in China’s urbanization process. In particular, the high number of high-rise building fires during 2008–2012 directly led to a research boom in this area. China was not involved in the research at the beginning of the theme. However, the contribution of Chinese scientists in this field triggered many countries to participate in this theme. From 2013 to 2015, there was a first phase of a research boom on this theme. From 2018 to the present, this theme received widespread academic attention;

(3) The most common technique for improving the flame retardancy of RPUF is the addition of additives with flame-retardant properties, of which expandable graphite is the most commonly investigated material. Halogen-free flame retardants and phosphorus-containing flame retardants are also very commonly used. In addition, some inorganic materials are also added to improve the flame-retardant properties of RPUF, such as graphene oxide, glass fiber, and a zeolitic imidazolate framework;

(4) The investigation of the flame-retardant mechanism is also an integral part of this theme. RPUFs with other flame-retardant additions have different flame-retardant mechanisms, but a comprehensive understanding of the flame-retardant properties of modified RPUFs is a challenging task. On the contrary, because RPUFs have strong industrial applications, their performance indicators are of more interest to researchers.

Meanwhile, based on the review of this theme, we believe that the following issues need to be investigated regarding flame-retardant RPUF:

(1) Different flame retardants have different effects on RPUF, and some of these choices produce very superior performance. However, RPUF is a highly consumed building insulation material, and the choice of flame retardant needs to be further considered in light of the cost challenge. Some new materials can be very effective flame retardants due to their excellent microstructure, but their cost is not suitable for practical application at this stage. However, it is valuable to investigate the flame-retardant mechanism of RPUF modification using these materials with characteristic microstructures;

(2) The investigation of the flame-retardant properties of RPUF in this theme was measured under ideal conditions rather than being imitated when used as building insulation. Therefore, the flame-retardant properties of these materials do not consider the effects that the building space can bring about. Only a limited number of papers focused on the combustion behavior of RPUF in different spatial orientations. Therefore, more future investigations should be focused on simulating the combustion behavior of RPUF in real situations;

(3) Although the addition of flame retardants can improve the flame-retardant effect of RPUF, the toxicological properties of the flame retardants themselves should also be taken into account. Since building insulation materials have a close relationship with human life, it is also important that flame retardants do not pose a health hazard. In addition, the decomposition products of the flame retardants themselves when they burn need to be investigated.

## Figures and Tables

**Table 1:** Top 15 cited journals on flame-retardant RPUF.

No.	Citation	Cited Journal
1	159	Journal of Applied Polymer Science
2	155	Polymer Degradation and Stability
3	96	Polymer
4	94	Polymers for Advanced Technologies
5	91	Industrial & Engineering Chemistry Research
6	84	Polymer International
7	69	Journal of Thermal Analysis and Calorimetry
8	68	Fire and Materials
9	68	European Polymer Journal
10	61	Polymer Composites
11	59	Progress in Polymer Science
12	57	Industrial Crops and Products
13	54	RSC Advances
14	49	Journal of Analytical and Applied Pyrolysis
15	49	Composites Science and Technology

**Table 2:** List of journals that have appeared in the co-occurrence network in the last two years.

Year	Journals	Standardizations/Reports
2021	Chemical Engineering and Processing; Current Opinion in Food Science; Chemical Papers; Applied Biochemistry and Biotechnology; Engineering Science; Catalysis Reviews; Applied Sciences; Advanced Science; Environmental Science & Technology Letters; Chemical Society Reviews; ES Energy & Environment; ACS Materials Letters; Energy Procedia; Additive Manufacturing; Reviews on Environmental Health; Journal of Cleaner Production; The Journal of Supercritical Fluids; Materials Science and Engineering: C; Waste Management; Materials Research Express; Molecules; ACS Omega	CEN/TC 127N1424
2020	Advances in Cement Research; Adsorption Science & Technology; Austral Ecology; Bulletin of Materials Science; Agronomy; Archives of Materials Science and Engineering; Advances in Polymer Technology; Advances in Materials Science and Engineering; Advances in Civil Engineering; Applied Mechanics and Materials; Plastics, Rubber and Composites; Applied Acoustics; Isi Bilimi Ve Teknigi Dergisi; Journal of Coatings Technology and Research; Journal of Porous Materials; Experimental Thermal and Fluid Science; Solar Energy Materials & Solar Cells; International Journal of Heat and Mass Transfer; Surface and Coatings Technology; Advances in Polymer Technology; Advances in Materials Science and Engineering; Advanced Powder Technology; Materials	ISO: 566012015; ISO: 8442014; ISO: 458922017; ISO: 1135742014; NISTIR:4664; ISO: 83011991; IOS: 8452009

**Table 3:** List of top 15 keywords for flame-retardant RPUF.

No.	Freq	Centrality	Keywords
1	54	0.19	Expandable graphite
2	44	0.22	Mechanical property
3	42	0.24	Behavior
4	39	0.10	Composite
5	34	0.10	Phosphorus
6	28	0.08	Ammonium polyphosphate
7	26	0.05	Fire behavior
8	26	0.09	Thermal degradation
9	21	0.08	Density
10	21	0.06	Degradation
11	20	0.11	Nanocomposite
12	18	0.04	Polyol
13	17	0.16	Flammability
14	14	0.03	Stability
15	13	0.04	Combustion

**Table 4:** Knowledge clusters in the theme of flame-retardant RPUF on keyword co-occurrences for each cluster.

Cluster ID	Size	Silhouette	Keywords	References
0	50	0.867	Expandable graphite; Mechanical property; Composite; Ammonium polyphosphate; Density; Degradation; Polyol; Flammability; Combustion; Thermal stability	[69,70,71,72,73,74,75,76,77,78,79,80,81,82,83,84,85,86,87,88,89,90,91,92,93,94,95,96,97,98]
1	25	0.872	Epoxy resin; Graphene; Thermal property; DOPO; Nanoparticle; RPUF; Silica; Formulation; Hypophosphite; Styrene	[47,99,100,101,102,103,104,105]
2	24	0.854	Stability; Oil; Phosphazene; Polypropylene Dimethylmethyl phosphonate; Montmorillonite; Fire hazard; Thermoplastic polyurethane	[46,76,106,107,108,109,110,111,112,113]
3	24	0.955	Polymer; System; Chemical; Property; Construction; Retardant behavior; Aluminum; Insulation	[114,115,116,117,118,119,120]
4	24	0.863	Firebehavior; Nitrogen; Fire retardant; Aluminum hydroxide; Nanocomposite foam; Layered silicate; Phosphorus containing compound	[70,78,79,121,122,123,124]
5	24	0.907	Behavior; Phosphorus; Performance; Particle; Mechanism	[33,71,73,76,79,84,85,109,125,126,127,128,129,130,131,132,133,134,135,136,137,138]
6	23	0.930	Nanocomposite; Combination; Polyphosphate; PU foam; Foam	[35,82,100,139,140,141,142,143,144,145,146]
7	22	0.852	Thermal degradation; Fire behavior; Fire retardancy; Halogen free; Thermal decomposition; Additive; Dimethyl methylphosphonate	[75,81,106,129,130,132,134,145,146,147,148,149]
8	13	0.913	Cyclotriphosphazene; Insulation material; Sample width; Thermal insulation; PMMA surface	[150,151]
9	13	0.940	Coating; Composite particle; Graphite; Thermal conductivity	[87,152,153,154,155]
10	12	0.910	Phosphate; Graphene oxide; Phase change material; Oxide	[68,71,83,156]
11	11	0.972	fabrication; Agent; Fire safety; Silicon	[47,157]

## Data Availability

Data sharing not applicable.
